# IL-10 is a critical negative regulator of macrophage activation syndrome

**DOI:** 10.1186/1546-0096-10-S1-A110

**Published:** 2012-07-13

**Authors:** Scott Canna, Katharine Slade, Michele Paessler, Portia Kreiger, Sheila Rao, Edward M Behrens

**Affiliations:** 1 A.I. Dupont Hospital for Children, Wilmington, DE, USA; 2 Children's Hospital of Philadelphia, Philadelphia, PA, USA; 3 University of Pennsylvania, Philadelphia, PA, USA

## Purpose

Macrophage Activation Syndrome (MAS, alternatively known as secondary Hemophagocytic Lymphohistiocytosis (HLH)) is a potentially life-threatening complication of many rheumatologic, infectious, and malignant diseases. It is characterized by the acute onset of disseminated intravascular coagulopathy and multi-system organ dysfunction in the setting of overwhelming inflammation. As a result of the poor understanding of disease pathogenesis, no consensus exists regarding how best to diagnose and treat this condition. Preliminary evidence in genetic forms of this disease (known collectively as primary HLH) suggests a critical role for IFNγ and CD8+ T-lymphocytes.

## Methods

We treated Wild-type (WT) and transgenic mice with the Toll-like Receptor 9 (TLR9) agonist CpG, IFNg-neutralizing antibody, and/or IL-10 receptor blocking antibody via intraperitoneal injection as discussed below.

## Results

WT mice treated with CpG develop symptoms suggestive of MAS, including pancytopenia, splenomegaly, hypercytokinemia, hyperferritinemia, hepatitis, and coagulopathy. Like primary HLH, CpG-induced MAS is also IFNg-dependent, as mice who are IFNg-deficient or in whom we have neutralized IFNg show a greatly abrogated phenotype. Accordingly, IFNg-deficient mice treated with CpG have minimal IL-10, suggesting an IFNg-dependent IL-10 response. In contrast, Rag knockout mice treated with CpG show an intact IFNg response but blunted IL-10 production, resulting in enhanced hepatitis. These data suggest the role of IL-10 in MAS is to regulate IFNg-mediated inflammation. To test this hypothesis, we treated mice with CpG and concomitantly blocked IL-10 signaling. These mice developed cachexia, more severe hepatitis & coagulopathy, worsened cytopenias, enhanced cytokinemia and numerous splenic hemophagocytic macrophages (HPCs). We also see expansion of the populations of IFNg-mRNA producing CD4+ & CD8+ T-cells in the liver when we neutralize IL-10 signaling when compared with CpG alone.

## Conclusion

Repeated TLR9 stimulation induces an MAS-like syndrome mediated by IFNg and negatively regulated by IL-10. IL-10 also appears to exert its negative effects on distinct lymphocytic targets with organ specificity. Future studies will refine the cellular sources and targets of IL-10 and IFNg, describe the mechanisms by which IL-10 works to modify TLR and/or IFNg signaling, and define the functional significance of HPCs. These insights will not only further our understanding of MAS, but all cytokine storm syndromes.

## Disclosure

Scott Canna: None; Katharine Slade: None; Michele Paessler: None; Portia Kreiger: None; Sheila Rao: None; Edward M. Behrens: None.

